# Profile of SARS-CoV-2

**DOI:** 10.1007/s00508-020-01763-1

**Published:** 2020-10-30

**Authors:** Franz X. Heinz, Karin Stiasny

**Affiliations:** Center for Virology, Medical University of Vienna, Kinderspitalgasse 15, 1090 Vienna, Austria

**Keywords:** COVID-19, Coronavirus, Origin and evolution, Vaccines, Antivirals

## Abstract

The recent emergence of a new coronavirus (severe acute respiratory syndrome coronavirus‑2, SARS-CoV-2) that is transmitted efficiently among humans and can result in serious disease and/or death has become a global threat to public health and economy. In this article, we describe some of the most important characteristics of this new virus (including gaps in our understanding) and provide a perspective of ongoing activities for developing virus-specific countermeasures, such as vaccines and antiviral drugs.

## Background

**Fig. 1:**
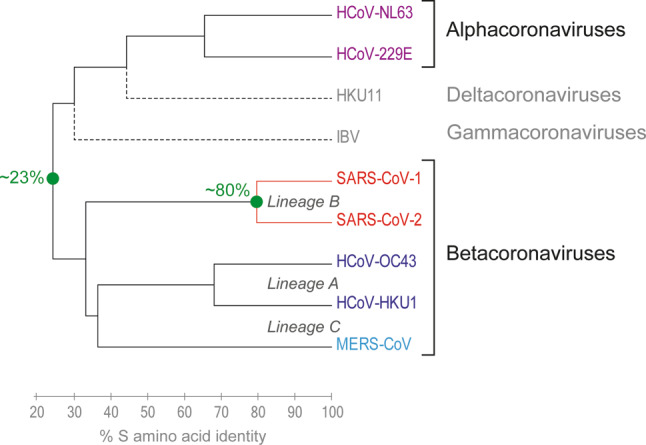
Dendrogram of coronaviruses assigned to the four genera of alphacoronaviruses, betacoronaviruses, gammacoronaviruses, and deltacoronaviruses (based on percentage amino acid sequence identity in the spike protein S). In betacoronaviruses, genetic lineages are indicated. HKU11 and IBV (infectious bronchitis virus) designate avian coronaviruses. *MERS-CoV* Middle East respiratory syndrome coronavirus

**Fig. 2:**
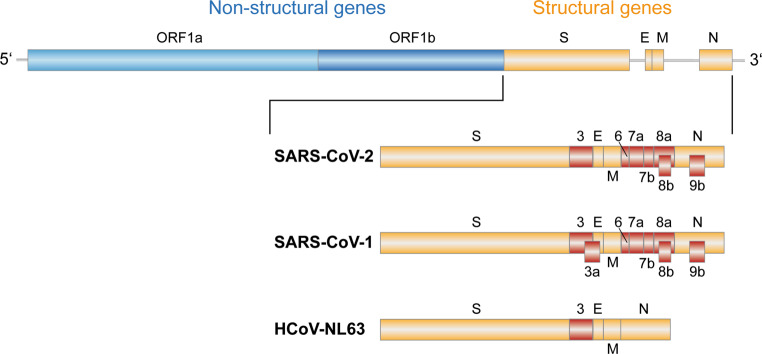
Genome organization of coronaviruses. Top panel: overall arrangement of nonstructural and structural genes. Lower panel: zoom of structural gene region, with accessory genes in *red*, as described by Xie et al. [94] for SARS-CoV‑2 and Forni et al. [2] for SARS-CoV‑1 as well as HCoV-NL63 [2]. *ORF* open reading frame. Designations of structural genes: *S* spike; *E* envelope; *M* membrane; *N* nucleocapsid

**Fig. 3:**
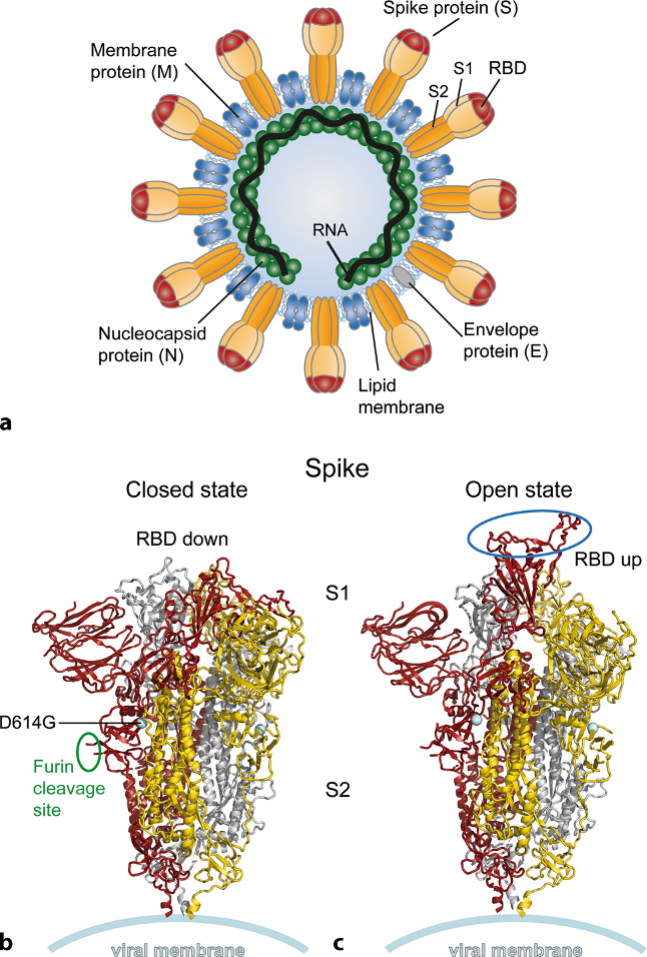
Structural organization of coronavirus particle and the spike protein S. **a** Schematic of virus particle. **b** Ribbon diagrams of the soluble S protein trimer. The three monomers are colored in *red*, *gold*, and *grey*. In the closed state, the receptor-binding domain (RBD) is in the ‘down’ conformation (protein data bank: PDB 6ZGI, [95]), and in the open state in the ‘up’ conformation (protein data bank: PDB 6ZGG, [95]), which can interact with the viral receptor ACE2 (angiotensin-converting enzyme 2). The interaction site is indicated by a *blue ellipse* in the *right panel*

Severe acute respiratory syndrome coronavirus‑2 (SARS-CoV-2) is the seventh coronavirus that has emerged as a human-to-human transmissible agent after zoonotic transfer from wild animals [1]. Coronaviruses are found globally in many animal species and are classified into four distinct genera (*Alphacoronavirus, Betacoronavirus, Gammacoronavirus* and *Deltacoronavirus*; Fig. 1), all sharing the same basic structural and genetic organization as well as replication strategy ([2, 3]; Figs. 2 and 3). Infections of humans and other mammals are caused by the alphacoronaviruses and betacoronaviruses, whereas gammacoronaviruses and deltacoronaviruses have birds as their primary hosts. In animals, coronaviruses cause a range of diseases (primarily respiratory and gastrointestinal infections) in a variety of species, resulting in serious consequences for the livestock industry [4]. Of the seven human coronaviruses (HCoV), four cause relatively benign respiratory infections and are distributed worldwide [3]. Two of them (designated 229E and Nl63) are alphacoronaviruses and have their likely origins in bats. The other two (designated OC43 and HKU1) are betacoronaviruses with a possible origin in rodents and/or cattle ([2, 3]; Fig. 1).

In 2002, the emergence of SARS-CoV‑1 in China and its global spread caused a first coronavirus-related human health crisis of international concern [5]. It became rapidly clear that this virus had jumped to humans from natural reservoirs in bats, likely through intermediate hosts such as civet cats [3]. Fortunately, human-to-human transmission could be interrupted completely by hygienic measures of epidemic control within a short period of time, and natural transmission of the virus came to an end by 2003 [6]. A second potentially deadly human coronavirus (Middle East respiratory syndrome coronavirus, MERS-CoV) emerged in 2012 in Saudi Arabia [7]. In this case, the source of transmission to humans was identified as infected dromedary camels. Although human-to-human transmission chains occurred and still do occur, they can be contained and do not result in widespread outbreaks. The risk of zoonotic infection and ensuing limited chains of infection, however, remains because of the enormous viral reservoir in dromedary camels, especially across the Arabic Peninsula and parts of eastern and northern Africa [8, 9]. Both SARS‑1 and MERS viruses are betacoronaviruses but belong to two different genetic lineages, B and C, respectively, distinct from lineage A which comprises HCoV-OC43 and HCoV-HKU1 (Fig. 1; [2]).

After the emergence of SARS-CoV‑1, efforts to identify natural reservoirs provided ample evidence for extensive circulation of SARS-like coronaviruses in bats with the potential of crossing species barriers and the emergence in humans [10, 11]. Corresponding publications were full of warnings that SARS-1-like emergences could happen again, unless contact to the natural reservoir of coronaviruses in bats and transmission to humans via intermediate hosts cannot be properly controlled [5]. Unfortunately, these warnings did not materialize in practice, and a very similar virus (now called SARS-CoV-2) emerged at the end of 2019 in Wuhan, China. From there, the virus rapidly spread globally and to date (25 October 2020) the pandemic has caused 43,341,451 documented cases of infection and 1,157,509 fatalities/deaths [12].

## Genome and virion structure

The genome of SARS-CoV‑2 is ~29,800 nucleotides in length [13] and has the characteristic features of coronaviruses in general (Fig. 2). They all have exceptionally large nonsegmented positive-stranded RNA genomes (ranging between ~26,000 and 32,000 bases [2]), which contain 3 sets of open reading frames (ORF) encoding three groups of proteins with different functions: 1. 16 non-structural proteins (nsp1 to nsp16) that include the viral RNA polymerase, proteases and other virus-specific enzymes involved in virus replication. 2. The structural proteins S (spike), E (envelope), M (membrane), and N (nucleocapsid) that are essential building blocks of virus particles (Fig. 3a) (some coronaviruses, e.g. HCoV-OC43 have a hemagglutinin-esterase protein as an additional structural component [2]). 3. A variable number of ORFs interspersed with the genes for structural proteins, encoding proteins that are produced in infected cells but are not essential for basic virus replication. These “accessory genes” can differ in number and sequence even among closely related coronaviruses and apparently contribute to the tuning of virus-host cell interactions to create an optimal environment for virus replication and transmission, e.g. by counteracting innate antiviral host responses [14]. The genetic mutation rate of SARS-CoV‑2 and coronaviruses in general is lower than that of other RNA viruses due to a proofreading activity in the viral RNA replication machinery [15, 16]. Nevertheless, coronavirus genomes have a high degree of plasticity and mechanisms that allow rapid virus evolution. These include a propensity for recombination (leading to the exchange of whole gene segments among different coronaviruses during double infections of the same cell in the same host) as well as the capacity for acquisition and losses of accessory protein coding genes, facilitating adaptive changes in addition to mutation [2]. Together with the structural flexibility of the spike protein, allowing adaptation to different cellular receptors with relative ease, these properties are the foundation for the propensity of coronaviruses for crossing species barriers and jumping hosts.

The SARS-CoV‑2 virus particle has the typical coronavirus morphology, with a diameter of ~80–120 nm [17]. Infectious virions are composed of a helical nucleocapsid (comprising the genomic RNA and multiple copies of the N protein) and a lipid envelope with three membrane-associated proteins, M (membrane), E (envelope), and S, forming the characteristic club-shaped projections at the viral surface (Fig. 3a). The spike protein has attracted special attention, because it provides the essential viral functions for cell entry (receptor binding and membrane fusion) and is the primary target of neutralizing and potentially protective antibodies [18]. It is therefore considered the most important and essential antigen component of vaccines (see section “Prospects for vaccines”). Each of the spikes is composed of three S protein subunits, and proteolytic cleavage of the S monomers into S1 (membrane-distal part) and S2 (membrane-associated part) by host cell proteases at two closely adjacent sites is required for infectivity [19]. The molecular architecture of S (Fig. 3b) reflects its dual function comprising a receptor-binding domain (RBD) in S1 and a spring-loaded fusion machinery in the membrane-associated S2 [19]. In its prefusion form the S‑trimer of SARS-CoV‑2 was found to oscillate between an open and a closed conformation with an up and down orientation of RBD (Fig. 3b), which only in the up conformation is capable of binding its specific receptor angiotensin-converting enzyme 2 (ACE2) [20–22]. Fusion of the viral membrane with a cellular membrane is required for the release of the genome into the cytoplasm. This process is driven by a triggered structural change of S2 from its metastable prefusion conformation into a stable postfusion form that is made possible by a combination of receptor binding and proteolytic cleavage between S1 and S2 and provides the energy for membrane fusion [19].

## Unique features of SARS-CoV-2 relative to SARS-CoV-1

The genome organizations of SARS-CoV‑1 and SARS-CoV‑2 are remarkably similar, with a 79% identity at the nucleotide level [23]. Both viruses bind with high affinity to the same receptor, ACE2, and are so far the only human coronaviruses in lineage B of betacoronaviruses (Fig. 1). Despite these similarities, distinguishing features were identified that are likely to contribute to the biological differences observed between the two viruses, including the significantly higher rate of subclinical and mild infections caused by SARS-CoV‑2, which makes control of virus spread currently so difficult. One of the major differences is in the spike protein, which—in contrast to SARS-CoV-1—has a polybasic cleavage site at the junction between S1 and S2 that is likely to facilitate cleavage activation by the cellular protease furin and/or other proteases [1, 20, 21, 24]. Such sequence elements are characteristic of highly pathogenic avian influenza viruses [25] and are also present in the human coronaviruses MERS-CoV, HCoV-OC43, HCoV-HKU1 [26]. Their impact on coronavirus transmissibility and pathogenesis has yet to be established. It is also noteworthy that the adaptive evolutionary changes in S that led to the recognition of ACE2 in SARS-CoV‑1 and SARS-CoV‑2, apparently followed different trajectories of mutations, with just one of the six critical amino acids in S that interact with ACE2 being identical between the two viruses [27–29]. The structural evidence thus suggests that these viruses acquired the ability to recognize the same receptor independently in a process of convergent evolution or, alternatively, diverged from a common ancestor a long time ago.

Potentially important differences are also present in some of the accessory proteins (Fig. 2). These include variations in ORF3b, which encodes an interferon antagonist in SARS-CoV‑2 that is more potent than its orthologue in SARS-CoV‑1 [30]. It is also remarkable that the sequence homology of ORF8 is exceptionally low between SARS-CoV‑1 and SARS-CoV‑2 (26% at the nucleotide level and 20% at the amino acid level), and also the gene products (8a and 8b) display significant differences [13]. It is currently unknown how precisely these differences shape the biological properties of the two viruses.

## Origin and evolution of SARS-CoV-2

In the aftermath of the SARS-CoV‑1 epidemic, a large number of related viruses were identified in bats, and conclusive evidence was obtained that the new human virus had originated from this animal reservoir, presumably via adaptation in other mammals, such as civet cats as intermediate hosts [10, 11]. One of these bat virus isolates (designated RaTG13) is so far the closest relative of the new SARS-CoV‑2, with approximately 96% identity in almost all genomic regions [31, 32]; however, RaTG13 is unable to recognize human ACE2 as a receptor and also does not have the polybasic cleavage site in S. Molecular clock analyses indicate that the two viruses are separated by more than 20 years of evolution [27], and it is therefore unlikely that this bat virus is a direct ancestor of SARS-CoV‑2, which has still to be identified. Interestingly, a virus isolated from pangolins was found to be able to interact with human ACE2 and to have an RBD which is much more closely related to that of SARS-CoV‑2, although the rest of the genome is significantly more different than that of RaTG13 [33, 34]. Specifically, the RBD of the pangolin virus contains all six key amino acid mutations required for binding to the ACE2 receptor, allowing infection of human cells. It has therefore been speculated that pangolins may have played a role as intermediate hosts in the evolution of SARS-CoV‑2 [34, 35], but conclusive evidence is still lacking. A second plausible scenario would be that a yet unknown bat virus with the capacity to use human ACE2 as a receptor was transmitted directly to humans and remained unidentified as a new human virus for a relatively long time. Further adaptive changes during this phase of cryptic spread could have improved transmission among humans until the virus became apparent at the start of the pandemic in Wuhan in December 2019.

One of the key questions in the current epidemic relates to possible further adaptive changes in SARS-CoV‑2 that could affect its transmission efficiency, disease severity, escape from immunity or other biological properties. Two mutations in circulating virus strains have raised special attention. One of them, a replacement of aspartic acid for glycine at position 614 in the spike protein (D614G) (Fig. 3b, left panel), has become dominant and is now found in almost all SARS-CoV‑2 samples worldwide [36]. Although in vitro models have provided evidence for a higher infectivity of the mutant virus [37], there is currently no definitive proof that it arose through a process of natural selection by increased transmissibility and/or immune escape rather than having become dominant as a result of a founder effect [38]. A variant with a deletion in ORF8 (∆382) was identified in Singapore and associated with a milder infection and less systemic release of pro-inflammatory cytokines [39]. As a contrasting example, a virus with a mutation in ORF3 that caused more efficient suppression of IFN‑I was found in two severely ill patients in Ecuador [30]. It will certainly be important to closely follow the emergence of possible adaptive mutations in circulating strains and to study how they might affect transmission and/or disease. The current situation, however, is not characterized by extensive variation but by a rather surprising stability of the virus. This may be due to the relatively low genetic mutation rate of this virus, which is ~2–6 times slower than that of influenza virus [16]. On the other hand, the lack of evidence for further adaptive changes might be an indication that the virus had not jumped to humans at the end of 2019 but significantly earlier and that adaptation to its new host after zoonotic transfer had already occurred in this early and cryptic phase of transmission.

## Pathogenesis and immune response

SARS-CoV‑2 is a cytopathic virus that is transmitted primarily by droplet infections and injures virus-infected cells in airway epithelial cells expressing the viral receptor ACE2 [40]. The disease caused by this new virus is called coronavirus disease 2019 (COVID-19) [41]. Early control of infection by innate immune responses is counteracted by several viral gene products [42, 43], and competition between virus and host defenses defines whether the infection is readily cleared (asymptomatic and mild respiratory infections) or proceeds to more severe forms including pneumonia, acute respiratory distress syndrome (ARDS), multiple organ failure and death. The life-threatening forms of COVID-19 are associated with the overproduction of immune cytokines and an uncontrolled systemic inflammatory response, frequently referred to as “cytokine storm” [44, 45]. In addition to early innate immune responses, infection also induces specific T and B cells that can potentially provide immunity against re-infection, at least for a certain period of time [46]. It is still incompletely understood to which extent the different effector arms of adaptive responses (such as cytotoxic T cells, helper T cells and antibodies) contribute to virus clearance and recovery in the acute phase of infection. It is also not yet clear whether T cell responses are only beneficial or contribute to the immunopathology [47].

The case fatality rate strongly increases with age, similar to infections with SARS-CoV‑1 and MERS-CoV, and may be linked (at least in part) to the phenomenon of immunosenescence that impairs both T cell and B cell responses in aged individuals [48, 49]. The fatality rate is strikingly higher in males than in females [50], which may be related to the enhanced expression of alleles on the X chromosome (including some Toll-like receptors involved in innate immunity but paradoxically also the receptor ACE2), stronger B and T cell responses in women, and/or different immunoregulatory functions of sex hormones [50, 51].

It is currently unclear to which extent the different components of adaptive immune responses induced by natural infection will prevent re-infection or at least severe disease and how long such immunity will last. Reinfections were shown to be quite common with the four seasonal human coronaviruses at 12 months after primary infection [52], and reinfection has also been demonstrated with SARS-CoV‑2 [53]. These data indicate that protective immunity after natural infection may not be long-lived and insufficient in certain cases. There is consensus, however, that a sufficiently high concentration of neutralizing antibodies, directed to the S protein and preventing virus entry into cells expressing the virus receptor ACE2, play an important role and are essential for conferring immunity [54]. It is unclear, however, which titer of neutralizing antibodies would be protective. Immunosenescence is certainly one factor that impairs antibody formation, but current evidence also indicates that antibody responses are lower in individuals with asymptomatic or mild forms of infection and decline more rapidly than in those with more severe disease [55]. Recent studies with COVID-19 patients indicate a virus-induced impairment of germinal center formation that is likely to hamper the generation of long-lived antibody responses [52, 56]. Immune responses may also be influenced quantitatively and qualitatively by pre-existing cross-reactive immunity. SARS-CoV-2-specific CD4 T cells have been found in 30–50% and CD8 T cells in 20% of healthy people with no evidence of SARS-CoV‑2 infection [55, 57, 58]. These cells were likely induced by past infections with one of the seasonal human coronaviruses and it remains to be investigated, how their presence can influence immune responses, the outcome of ongoing infections with SARS-CoV‑2, and postinfection immunity.

## Prospects for vaccines

Development of vaccines against SARS-CoV‑2 has proceeded with an unprecedented pace and breadth [54, 59, 60], although it is still hampered to a certain extent by the lack of an accepted in vitro correlate of protection. Currently, the World Health Organization (WHO) lists 198 experimental vaccines in preclinical or clinical development (https://www.who.int/publications/m/item/draft-landscape-of-covid-19-candidate-vaccines, as of 19 October 2020, accessed on 28 October 2020). All technologies available to date are being exploited, including inactivated whole virus vaccines, protein subunit and virus-like particle vaccines, nucleic acid vaccines (DNA and RNA), different kinds of viral vector vaccines, and live attenuated vaccines. Since there is consensus that the induction of neutralizing antibodies (directed to the S protein) is the key to the success of vaccine-induced immunity, virtually all current experimental vaccines build on this protein (or part of it) as a key antigen [55].

At the date of submission of this article, ten vaccine candidates are the most advanced and currently evaluated in phase 3 clinical trials (https://www.who.int/publications/m/item/draft-landscape-of-covid-19-candidate-vaccines, accessed 2 October 2020). These front runners include three betapropiolactone-inactivated whole virus vaccines grown in Vero cells (Sinovac Biotech, Beijing, China and Sinopharm, Beijing, China), two mRNA vaccines (Moderna [Moderna, Cambridge, MA, USA]/NIAID and Biontec [BioNTech, Mainz, Germany]/Fosun Pharma [Fosun Pharma, Shanghai, China]/Pfizer [Pfizer, New York City, NY, USA]), four Adenovector vaccines (University of Oxford/Astra Zeneca [Astra Zeneca, Cambridge, UK]; Can Sino Biological [CanSino Biologics, Tianjin, China]; Gamaleya Research Institute [Gamaleya Reseearch Institute, Moscow, Russia]; Johnson and Johnson [Johnson & Johnson, New Brunswick, NJ, USA]/Janssen [Janssen Pharmaceutica, Beerse, Belgium]), and one recombinant S protein subunit vaccine (Novavax [Novavax, Gaithersburg, MD, USA]). mRNA vaccines contain the coding sequence of the full length S protein and in some instances modifications for maintaining a native protein structure [20, 21] or specific nucleotide modifications for balancing innate immune responses [61, 62]. Adenovector vaccines contain defective adenovirus particles in which part of the genome is replaced by the coding sequence of the SARS-CoV‑2 S protein. In some vaccines, these constructs contain mutations like those engineered into the mRNA vaccines for stabilizing the S protein. The recombinant adenovirus particles can enter cells and express the S protein in a single round of infection. Three of the four experimental vaccines use human adenovirus vectors (based on Adeno 5 and/or Adeno 26), which have the drawback that pre-existing adenovirus immunity can impair their immunogenicity [55]. In contrast, University of Oxford/Astra Zeneca have made use of a chimpanzee adenovirus, for which almost no immunity exists in humans [63]. Nevertheless, vaccination-induced immunity against the vector can still potentially impair immune responses to booster vaccinations, a problem that may be solved by using vaccination schedules with alternative adenovirus serotypes as vectors [54].

In all instances, results from phase 1 and 2 clinical trials have shown the induction of B and T cell responses together with the formation of neutralizing antibodies [54]. The extent of side reactions, although usually mild or moderate and not serious, was quite common with some of the vaccines [54], and their tolerance in mass vaccination campaigns may become an important criterion for acceptance by the population. Since validated in vitro correlates of protection do not yet exist, a true snapshot of vaccine-induced prevention of disease will only become available from the evaluation of phase 3 clinical trials. Some of the next important questions will then be how long such protection lasts, how immunogenic the vaccines are in the older and most vulnerable population, whether the vaccines can generate herd immunity, and whether booster vaccinations are necessary to maintain long-term protection. Considering the plans for vaccinating millions or even billions of people, special attention will also have to be paid to indications of potentially serious consequences of dysregulated immune responses that would occur late (e.g. upon natural infection), such as antibody-dependent enhancement (ADE) of infection [64–66].

The performance of the vaccines in currently ongoing phase 3 trials cannot be predicted but differences among the candidates are to be expected—both with respect to protection and side reactions—and suboptimal rates of both parameters are possible. In this way, the performance of the front runners will determine the fate of the second line of candidates pushing forward in the race to a COVID-19 vaccine. If expectations were too optimistic and results obtained with some of the front runners are disappointing, windows of opportunity will open for an arsenal of alternative developments in progress [54, 59] (https://www.who.int/publications/m/item/draft-landscape-of-covid-19-candidate-vaccines, accessed 2 October 2020) These include subunit vaccines with S proteins stabilized in their prefusion conformation in combination with potent adjuvants, use of the RBD only as an immunogen instead of the whole S protein [67, 68], other rationally designed immunogens [69], other (non-Adeno) vector vaccines including replication-competent vectors [55, 70], self-amplifying RNA vaccines [71], live-attenuated vaccines [55], DNA vaccines [72], and intranasally applied vaccines with the potential to induce local immunity at the site of virus entry [73]. At the current stage of investigation, it is uncertain, whether and to what extent a COVID-19 vaccine can induce durable protection from infection, or more importantly, from severe disease. We also do not yet have information as to the effect of pre-existing cross-reactive coronavirus immunity on vaccine performance, an important aspect to be considered in evaluations of phase 3 clinical trials. One of the most crucial questions relates not only to individual protection, but also to which extent vaccination will be able to confer herd immunity, the death toll of which would be very high when allowed to be induced by natural infection. A positive aspect is certainly the fact that SARS-CoV‑2 appears to be genetically quite stable, and there is currently no evidence for antigenic drift [38, 74], which would make vaccine development even more challenging.

## Specific antivirals

The dramatically increasing load of public health systems with patients suffering from severe COVID-19 was paralleled by an intensive search for antivirals that would specifically inhibit virus replication and thus extend treatment options. As a first and most rapid approach, attempts were made to repurpose existing drugs that had already been licensed for the treatment of other infectious diseases [75, 76]. Two of the most prominent substances studied in this context were hydroxychloroquin (used for the prophylaxis and therapy of malaria) and remdesivir, which had been developed as a specific treatment of Ebola disease and used for the treatment of SARS-CoV‑2 infections [75, 77]. Unfortunately, results of clinical application and trials did not meet the high expectations. Hydroxychloroquin was shown to have no beneficial effect for COVID-19 patients and therefore cannot be recommended for the treatment of SARS-CoV‑2 infections [78, 79]. Results from clinical trials with remdesivir are somewhat conflicting and revealed a modest effect in some treatment schedules [80]. Additional studies will be needed to resolve existing uncertainties of the incremental benefit achieved by adding remdesivir to standard treatments of COVID-19 patients [80]. More specific avenues of drug development may be opened by the determination of high-resolution structures of the SARS-CoV‑2 RNA-dependent RNA polymerase (RdRp) [81] and the main viral protease (Mpro) [82], responsible for the specific cleavage of protein precursors resulting from primary translation of the viral genome (Fig. 2). Both of these enzymes play a pivotal role in the viral life cycle, and knowledge of their atomic structures can inform the specific design of highly potent inhibitors of virus replication.

Another option for interfering with virus replication is to block interaction of S with its cell entry receptor ACE2 (Fig. 3). Inhibition of receptor binding is one of the classical modes of antibody-mediated virus neutralization, and convalescent plasma therapy has shown some promise in the treatment of COVID-19 patients [83, 84]. In addition, a number of potently neutralizing human monoclonal antibodies have been developed and are currently investigated in the context of phase 3 clinical trials as a more advanced approach compared to plasma therapy (https://www.nih.gov/news-events/news-releases/clinical-trials-monoclonal-antibodies-prevent-covid-19-now-enrolling).

The interaction of S and ACE2 may alternatively also be blocked by applying an excess of a soluble form of the receptor itself, produced as a recombinant protein [85]. This molecule was shown to inhibit virus replication in human cells and organoids [86], although its virus-neutralizing activity was not as high as that of the most potent monoclonal antibodies described so far [87–89]. It has to be considered, however, that the application of ACE2 may have additional beneficial effects by rescuing cellular ACE2 activity and thus preventing injury to the lungs [90]. Results from ongoing phase 2 clinical trials in Europe (EudraCT Number: 2020-001172-15) with recombinant soluble human ACE2 for the treatment of COVID-19 patients are pending.

## Conclusion

The global threat by the emergence of SARS-CoV‑2 as a new human pandemic virus has not only led to drastic public health measures for mitigating virus spread, but was also accompanied by an immediate and impressive response of basic and applied research, aiming to understand and combat this new virus. Substantial advances have already been achieved in all relevant areas, including genetics and structure of the virus, virus diagnostics, viral pathogenesis, public health measures of infection control, clinical treatment, and vaccine development. Despite the successes in all of these areas, science has still to answer a number of crucial and difficult questions. Some of the most eagerly expected answers are certainly to the questions of whether protection and herd immunity can be achieved by vaccination, how long vaccine-induced immunity will last, which immediate side reactions have to be tolerated, and whether long-term adverse events will be encountered.

The highly divergent spectrum of SARS-CoV-2-induced disease—ranging from a high proportion of asymptomatic or mild courses to life-threatening and fatal forms of infection—has put some general black boxes of infectious diseases into the spotlight, which are of special medical interest. The large number of subclinical or mild infections is not unique to SARS-CoV‑2 but is frequently encountered with some of the most prominent viruses and represents more the rule than the exception. As examples, poliovirus infections result in poliomyelitis in only 1 of 100 to more than 1 of 1000 individuals infected with the virus [91], and West Nile virus infections lead to central nervous system disease in only about 1 of 150 human infections [92]. Many factors are believed to contribute to these differences in individual disease permissiveness and progression, including dose of infecting virus, genetic factors, effectiveness and balance of immune responses, physiological factors and their combinations. The newly made experiences with COVID-19 underpin our lack of a detailed understanding of pathogenesis of virus infections in general and will certainly provide a new impetus for research into this fascinating area of virus-host interactions.

Finally, we certainly do not want this to happen again. The risk of a new outbreak, however, remains real, considering the vast number of coronaviruses circulating in bat populations and their potential of zoonotic transmission and adaptation to humans [11, 93]. Investigations of the natural reservoir will therefore be of paramount importance, as well as the further development of strategies to avoid dangerous contacts at the animal-human interface facilitated by illegal and legal trade of wildlife.
